# End-of-Life Planning in Aging Couples: A Comparison of Gay, Lesbian, and Heterosexual Marriages

**DOI:** 10.1093/geroni/igaf122.132

**Published:** 2025-12-31

**Authors:** Yiwen Wang

**Affiliations:** University of Central Florida, Florida, United States

## Abstract

Research on end-of-life planning within marriage has predominantly focused on heterosexual couples, overlooking the experiences of sexual minority couples. However, evidence suggests that many processes considered normative in different-sex marriages unfold differently in same-sex marriages. This study employs a minority stress perspective to examine how heterosexual, gay, and lesbian married couples engage in both informal (e.g., discussions about future care and end-of-life preferences) and formal (e.g., legal arrangements through living wills and other documents) end-of-life planning. I leverage dyadic data from the Health and Relationships Project (2015-2022) to compare the responses of men and women in same-sex and different-sex marriages. Using the Actor- Partner Interdependence Model (APIM), I assess how advance care planning are shaped by both spouses’ health, past health crises, dementia worries, relationships with family members, and experiences of discrimination. Findings suggest that gay and lesbian couples are more likely than heterosexual couples to engage in both informal and formal end-of-life planning, with these disparities largely driven by health concerns and fears of discrimination. These results highlight the need to consider the rapidly changing legal and social landscape and its implications for end-of-life planning among same- and different-sex couples.

